# Forcible Straightening of Carious Spines

**Published:** 1900-09-22

**Authors:** 


### FORCIBLE STRAIGHTENING OF CARIOUS SPINES.

With his usual vigour of thought and of expression, Mr. Edmund Owen went on to state his views on the practice of forcibly straightening the spine in cases of tuberculous disease with deformity. "Though I should be grieved to stand in the way of surgical advancement," he said, "I do not mind getting in the road and temporarily impeding traffic while we are taking time to consider the route, and are assuring ourselves that the stream of surgical progress is going in the right direction. My opinion is that the deformity of Pott's disease does not lend itself to operative treatment; that forcibly to interfere with it is to thwart Nature in her good attempts at effecting a curative consolidation in her own way. . . . I think, further, that in a short time we shall hear very little about the method. This is what I *think*; but I am absolutely *sure* of this—that if a child of my own had an angular deformity of its spine no person on earth should be allowed roughly to meddle with it. This is the only trustworthy way of testing one's opinion concerning the therapeutic value of speculative methods of treatment." There is, however, a small group of cases for which Mr. Owen thinks that forcible rectification of the angular deformity may perhaps eventually be found suitable, namely, a certain few of those in which pressure by bone, or by organising inflammatory deposits, has taken place upon the anterior surface of the cord, so that the patient has lost the power of voluntary movements in the lower extremities.

